# Facile promoter deletion in *Escherichia coli *in response to leaky expression of very robust and benign proteins from common expression vectors

**DOI:** 10.1186/1475-2859-8-8

**Published:** 2009-01-26

**Authors:** Martin Kawe, Uwe Horn, Andreas Plückthun

**Affiliations:** 1 Biochemisches Institut, Universität Zürich, Winterthurerstrasse 190, CH-8057 Zürich, Switzerland; 2 Leibniz Institute for Natural Product Research and Infection Biology – Hans-Knöll-Institute, Beutenbergstrasse 11a, 07745 Jena, Germany

## Abstract

**Background:**

Overexpression of proteins in *Escherichia coli *is considered routine today, at least when the protein is soluble and not otherwise toxic for the host. We report here that the massive overproduction of even such "benign" proteins can cause surprisingly efficient promoter deletions in the expression plasmid, leading to the growth of only non-producers, when expression is not well repressed in the newly transformed bacterial cell. Because deletion is so facile, it might impact on high-throughput protein production, e.g. for structural genomics, where not every expression parameter will be monitored.

**Results:**

We studied the high-level expression of several robust non-toxic proteins using a T5 promoter under *lac *operator control. Full induction leads to no significant growth retardation. We compared expression from almost identical plasmids with or without the *lacI *gene together in strains expressing different levels of LacI. Any combination without net overexpression of LacI led to an efficient promoter deletion in the plasmid, although the number of growing colonies and even the plasmid size – all antibiotic-resistant non-producers – was almost normal, and thus the problem not immediately recognizable. However, by assuring sufficient repression during the initial establishment phase of the plasmid, deletion was completely prevented.

**Conclusion:**

The deletions in the insufficiently repressed system are caused entirely by the burden of high-level translation. Since the *E. coli *Dps protein, known to protect DNA against stress in the stationary phase, is accumulated in the deletion mutants, the mutation may have taken place during a transient stationary phase. The cause of the deletion is thus distinct from the well known interference of high-level transcription with plasmid replication. The deletion can be entirely prevented by overexpressing LacI, a useful precaution even without any signs of stress caused by the protein.

## Background

Microorganisms have not evolved to produce single proteins in large amounts. Nonetheless, they can be programmed to do so, and this is one of the foundations of modern biological research and biotechnological applications. Yet, some recombinant proteins can be toxic to the host, either by interference with a cellular function or by their physical properties, e.g., proteins that interact with the membrane, and are thus strongly selected against (see below). However, we are not concerned with such proteins in the present study. Even proteins that are completely "benign" to the host are a metabolic burden when overexpressed in very large amounts [1]. Depending on the expression system, this could in principle be an effect of plasmid replication, transcription or translation. While the first two have been well studied (see below), the effects of extremely high translation by itself have not been directly addressed.

We show here that very efficient translation can itself be a serious problem directly after transformation, and that in the absence of sufficient repression, *Escherichia coli *finds surprisingly efficient ways of promoter deletions, leading to the exclusive growth of non-producing, yet antibiotic-resistant cells. We also show that this problem can be controlled and completely prevented by securing tight control of the repressible promoter system by providing the repressor in sufficient amounts.

In this article, we do not wish to put emphasis on how to control this problem, as this is readily done by overproducing the repressor protein, but on the fact that it can go easily undetected. While a thorough optimization of all expression parameters is important in large scale fermentation, especially for recurring industrial production, and thus they will be measured, for smaller scale batch cultures used in research, this is not the case, as it is generally sufficient that the process is robust and high-yielding. Under such high-throughput conditions, it is possible, therefore, that this facile promoter deletion might go undetected.

The strong overexpression of recombinant proteins is well known to lead to different stress reactions and interferes with cellular processes in many ways [2-4], which may finally lead to a "viable but non-culturable" cell state of the host organism [5]. The reason for this stress response and the adaptation of metabolic activities and cellular physiology under conditions of high-level recombinant protein production can in principle be attributed to the burden of the production of the plasmid, the mRNA and the protein product.

The metabolic burden for plasmid maintenance is by itself usually considered negligible [6], even though many differences can be observed when comparing plasmid-bearing cells with their plasmid-free counterparts [7], with size and copy number being important parameters [8-10]. Therefore, plasmids with runaway replication [11] are usually not preferred; at least, runaway plasmid replication should be inducible. Most expression vectors have thus high but stable copy numbers.

High-level transcription of the gene of interest, on the other hand, can be a serious problem, but not primarily because of consuming resources. Rather, when this high-level transcription interferes with plasmid replication [12-14], it decreases the plasmid copy number, which influences both antibiotic resistance and final product yield. This can be controlled both by placing an efficient transcription terminator at the end of the cassette, and/or by choosing the same direction of transcription as the unidirectional ColE1 replication fork. Thus, many modern expression vectors have been designed to avoid problems at the level of replication and transcription, and the system used here is no exception (see below), as transcription of the protein of interest is isolated by several transcription terminators from replication and other genes.

More severe perturbations of cellular metabolism are in general encountered by the high-level production of the plasmid-encoded protein itself. The response of the cell to such "translational stress" can be manifold, and will usually be a function of the recombinant protein itself: e.g. poorly folding proteins can trigger the heat-shock response of the cell [15,16] and toxic proteins (e.g. enzymes with deleterious activity) may lead to increased mutational alterations of their respective genes [17,18]. However, the production even of benign proteins is an enormous stress to the cell when under control of extremely strong promoters and Shine-Dalgarno sequences [19], and it can interfere with cellular processes in many ways [20,21]. Here we report that this stress is not necessarily directly detectable, since the promoter is rapidly eliminated, leading to the growth of cells in normal numbers which, however, do not produce the protein of interest.

We observed this surprisingly easily generated promoter deletion in several *E. coli *strains (DH5α; RV308; SB536) in response to massive overexpression of very robust proteins which neither by their protein function nor their properties show any obvious toxicity, but only as a consequence of the resources needed to synthesize them. This promoter deletion can go unnoticed up to the point of detecting a complete lack of expression in normally growing cultures. We reiterate that this deletion event can be entirely prevented and protein overproduction be sustained by overexpressing the LacI protein either from the bacterial chromosome or from the expression plasmid.

## Results and discussion

### Expression plasmid design and experimental system

We used a plasmid expression system with well established components, closely analogous to the widely employed pQE30 backbone (a commercial vector from Qiagen). It consists of the strong inducible T5 promoter under control of two *lac*-operator [22] sequences in series, the ORF of the protein of interest, followed by two terminators in series (a T7 transcription terminator and a strong *rrnB *t_1 _transcription terminator [23]), a ColE1 origin of replication (as present in pBR322, but without the *rop *gene [24]), the β-lactamase gene under its own promoter to confer resistance to ampicillin with the strong transcription terminator t_0 _of phage λ, and, facultatively, the *lacI *gene coding for the *lac *repressor, which is under control of the *lacI*^*q *^promoter and terminated by the strong t_HP _terminator [25], for tight regulation of the operon. The expression vectors used are called pMPAG77 (without *lacI *gene cassette), and pMPAG6 (carrying a *lacI *gene cassette under the *lacI*^*q *^promoter, this being the only difference to pMPAG77) (see Additional file 1).

As test proteins we used four different proteins which have in common that they are monomeric, very soluble and expressible in very high amounts in *E. coli*: the Designed Ankyrin Repeat Protein (DARPin) G3, an ErbB2-binder [26], the unselected DARPin E3_5 [27], coat protein D (pD) form phage λ [28] and *E. coli *maltose binding protein (MBP [29]).

As expression hosts 5 different *E. coli *strains were used (see Methods section for genotypes), two of which are overexpressing the *lac *repressor under the *lacI*^*q *^promoter, either on an F-plasmid (XL1 blue F' [30]) or integrated in the chromosome (DH5αZ1 [31]; which is DH5α *lacI*^*q*^*tetR*^+^), or three strains without the *lacI*^*q *^genotype (RV308 [32], DH5α [33] and SB536 [34]. For simplicity, we refer to the strains as *lacI*^*q*+ ^(XL1 blue F', DH5αZ1) and *lacI*^*q*- ^(RV308, DH5α, SB536).

### Expression of well expressible proteins in a lacI^q- ^background

When we transformed *lacI*^*q*- ^strains with the expression plasmids not containing a *lacI *gene, only a slight reduction in transformation efficiency was observed on ampicillin containing plates, yielding about 0.5–0.8 times the colony number of that obtained when a *lacI*^*q*+ ^stain was transformed with the same plasmids, or when the corresponding plasmid containing the *lacI *gene was used in either type of strain. Since this reduction in colony number was still in the range of variation for different preparations of competent cells, it would have stayed unnoticed without the respective controls. Only the coding sequence of protein D in the vector without *lacI *gene gave a stronger reduction in colony number in the *lacI*^*q*- ^strains than in either of the *lacI*^*q *^controls. It should be noticed that no outgrowth in liquid media after transformation and only a short incubation on ice was performed. Instead, cells were directly plated on solid media. It is, therefore, very unlikely that a preexisting deletion within the plasmid pool was enriched such that it could explain the nearly equal transformation efficiencies under conditions of incomplete or complete repression of protein expression. Furthermore, the plasmid used for test-transformation of *lacI*^*q*- ^strains had always been prepared from *lacI*^*q*+ ^strains.

**Figure 1:**
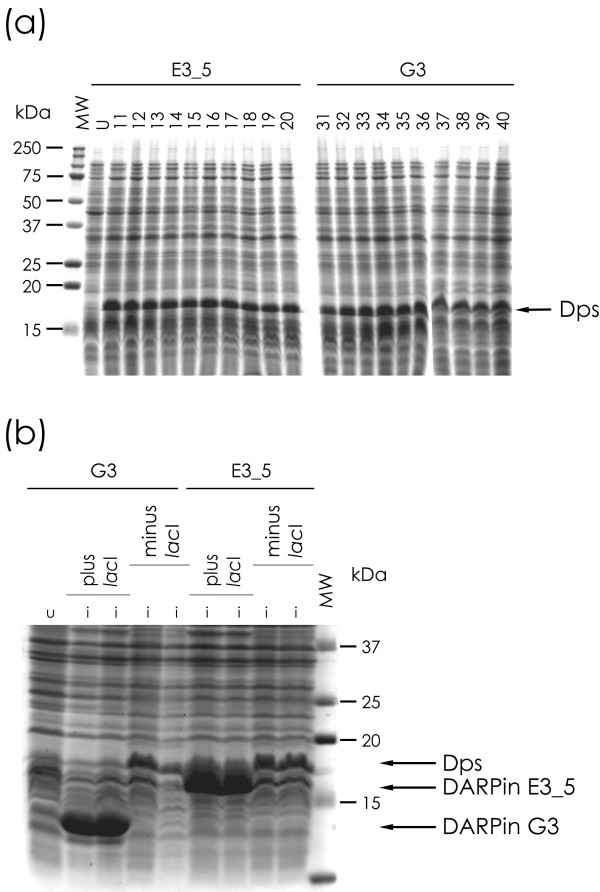
**Small scale expression tests of individual clones in a *lacI*^*q*+ ^or *lacI*^*q*- ^background**. (A) Expression test of 10 clones each of the DARPins E3_5 and G3 in *E. coli *RV308 (*lacI*^*q*-^) from expression vector pMPAG77 (*lacI*^*q*-^). *MW*, molecular weight marker; *U*, uninduced, *number*, clone number induced with IPTG. No protein band of the correct size in 15% SDS-PAGE analysis could be observed. Instead, a prominent band of ~19 kDa of a host protein was observed upon induction with IPTG (black arrow), which was identified as *E. coli *Dps. (B) Expression of DARPins E3_5 and G3 (two individual clones each) in *E. coli *DH5α (*lacI*^*q*-^) can only be observed in 15% SDS-PAGE analysis if LacI is provided by the expression plasmid. *U*, uninduced; *I*, induced with IPTG.

Small scale protein expression tests of single clones of the *lacI*^*q*- ^strains, transformed with expression plasmids not encoding the *lacI *gene, resulted in normal growth, but no expression at all for each of the tested proteins (Figure 1(a), (b)). Lack of expression was additionally confirmed by a western blot analysis of the RGS-His-Tag of our test proteins (data not shown). In contrast, normal, very high protein expression for all proteins tested could be observed for single clones originating from colonies of the *lacI*^*q*- ^strains transformed with expression plasmids encoding *lacI*, which was also confirmed by western blot (Figure 1(b) and data not shown). Normal high expression was also observed in single clone protein expression analysis of *lacI*^*q*+ ^strains, no matter whether they were transformed with the plasmids encoding *lacI *or plasmids not encoding *lacI *(data not shown).

To investigate whether the plasmid may have become mutated, we isolated the DNA of the non-producers from *lacI*^*q*- ^*E. coli *strains and introduced them into *lacI*^*q*+ ^strains. Such retransformation did not lead to any protein expression of the respective test proteins upon induction with IPTG, indicating that the non-producing phenotype had become encoded on the plasmid. The same type of plasmid leads to extremely strong expression when isolated from a *lacI*^*q*+ ^background.

PAGE-analysis of whole cell lysates of small scale protein expression tests with *lacI*^*q*- ^strains harboring plasmids not encoding *lacI*, while not showing any trace of the protein of interest, frequently led to the appearance of a prominent protein band of around 19 kD (Figure 1(a)). N-terminal sequencing of the respective excised band and subsequent BLAST-search identified this protein as Dps (DNA protecting during starvation protein; SWISSPROT: P0ABT2; PDB-ID: 1L8H), which is expressed by most bacteria at high levels under various stress conditions (e.g. heat, depletion of nutrients, oxidative stress) to protect its DNA [35].

### Sequence analysis of expression plasmids

**Figure 2:**
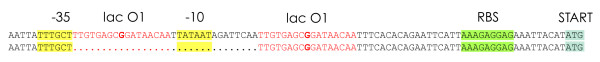
**Sequence Analysis**. Sequence of the promoter/operator region, identical in pMPAG6 (*lacI*^*q*+^) and pMPAG77 (*lacI*^*q*-^) (top line), and alignment with the deletions (bottom line) isolated from *lacI*^*q*-^*E. coli *strains harboring the respective *lacI*^*q*- ^expression vector: identical sequences were found in at least 6 independent clones of each construct protein. The -35 and -10 regions are indicated by yellow boxes; the two *lac O1 *operator sites are marked by red letters (length defined as the LacI contact residues seen by NMR [36], with each symmetry center in bold). The ribosomal binding site (RBS) is highlighted in green and the start codon in light blue, respectively.

To determine the reason for this unexpected complete failure of protein expression we analyzed the sequences of several clones of our expression plasmids for our different test proteins. Plasmid preparations not encoding *lacI *for the various test proteins were either prepared from *E. coli *RV308 or DH5α (both are *lacI*^*q*-^). Sequence analysis revealed that, independent of the examined test proteins (DARPins E3_5 or G3) a 32 bp fragment of the respective expression vector including the -10-region and one of the homologous operator-regions had been deleted when the plasmid preparation originated from a strain not overexpressing LacI (Figure 2) [36], independent of the test protein. The remaining vector backbone including the CDS of the protein of interest was correct in all cases and did not show any mutational variability. Thus, this deletion in the operator/promoter region of our expression plasmids, which is only occurring when both the strain is *lacI*^*q*- ^*and *the plasmid does not encode *lacI*, is the reason for the complete lack of protein expression under such circumstances.

### Transcription vs. Translation

To examine whether this promoter deletion was caused by cell stress originating from the level of transcription of the very strong T5 promoter [22] or from the level of translation, we constructed a pMPAG77-expression plasmid variant (not encoding *lacI*) which contained a stop codon in the ORF after the fifth amino acid position. By this measure we are recruiting a similar amount of the transcription machinery to this altered construct to produce mRNA as with the full length construct. While the translating ribosomes will likely protect the mRNA from degradation [37], they would not be expected to influence its initial biosynthesis rate. Therefore, if high-level transcription interfered with plasmid replication or if there was a metabolic burden due to massive RNA biosynthesis, this problem would remain when translation is abbreviated to a pentapeptide by an early stop codon.

Transformation rates of *lacI*^*q*- ^cells harboring this altered construct turned out to be as high as with the full length construct in a *lacI*^*q*+ ^strain background, and higher than with the corresponding construct lacking the stop codon in a *lacI*^*q*- ^background (data not shown). Sequence analysis of individual clones of this construct with the inserted stop codon prepared form *lacI*^*q*- ^strains did not show the deletion described above, nor did they have any other mutation in the gene (data not shown). This observation also strongly argues against the objection that such a deletion is already present in the starting plasmid pool. If this were true, it should be observed with the shortened construct as frequently as with the full length product, which is obviously not the case.

Doubling rates after induction were essentially indistinguishable between cells expressing the full proteins or the truncated versions. This observation is very distinct from the complete cessation of growth usually observed when the expression of "toxic" proteins is induced, e.g. some integral membrane proteins. This shows that, while high level protein expression is a resource problem, *E. coli *can handle it very well once the plasmid is established, at least during the few doublings of a batch culture.

Taken together, these observations suggest that the observed promoter deletions occur in response to massive overproduction of the respective protein and not due to problems at the transcriptional level, neither due to transcription/replication interference nor at the RNA resource level. The deletion event disappears once a stop codon is introduced shortly after the start codon of the ORF of our test proteins. This observation strongly argues that the deletion occurs in response to resource problems on the translational level and not on the transcriptional level. The deletion occurs in the present plasmids, which are very closely analogous to the widely used expression vector pQE30 (Qiagen) in strains lacking the *lacI*^*q *^phenotype (RV308, DH5α, SB536). This vector has been shown, otherwise, to be very robust and not to show any interference of transcription and replication. In our vector the strong transcript is isolated by strong terminators in both directions, and similarly the origin of replication is protected.

### Potential mechanism of deletion

The exact underlying mechanism for deletion of part of the promoter region still needs further investigations. The *E. coli *cells exploit the homology between the two *lac *operators, but the deletion is only observable under resource stress from translation. In the presence of sufficient LacI, this system is extremely well repressed [12]. After transformation, the plasmid starts from a single copy and is replicated until it reaches its steady state copy number, perhaps offering multiple opportunities for mutagenesis. It is likely that the resource stress and thus the selection operates at this time.

Deletions between short tandem repeats are thought to occur predominantly by "replication slippage" during replication and are favored when repeats are in close proximity, as they need to be present in the same replication fork [38-40]. As replication slippage is RecA-independent, this is consistent with our observation that the deletions also occur in *E. coli *DH5α, which has a *recA1 *phenotype.

While it has been proposed that these events are triggered by stalled replication forks [38,39], and one might suspect strong transcription to somehow lead to this situation, we note that the deletions were not found when a stop codon was introduced into the main transcript of an otherwise identical vector. We can, at present, not distinguish whether the intrinsic replication error is still rare when high level translation occurs and the deletion product is only strongly selected by the translational resource limitation or whether the resource limitation also increases the intrinsic frequency of this misalignment event. There has been a long standing controversy as to what degree selective stress induces mutations or only affects the relative growth advantage of preexisting mutants [41-44]. Our experiments cannot resolve this controversy, and different mechanisms may be operative, but we note the obvious signs of serious stress evidenced by the accumulation of the Dps protein [35]. Dps is a sign of early stationary phase [35], and thus the mutations are somewhat reminiscent of the observations of stationary phase mutagenesis described by Balbinder [45,46], even though the underlying mechanism is probably very different.

## Conclusion

As a practical consequence of the observations described here, we recommend using *fully *repressible systems for massive routine protein overproduction, even if none of the expressed proteins show signs of growth retardation or other evidence of toxicity. For strongly expressed, stable and soluble proteins, resources will not be recycled by protein degradation, and the problem may be even more acute. The existence of resource limitation may not be immediately apparent, as there are no obvious effects on growth or colony number after transformation, since deletion is surprisingly frequent. For *lac *operator controlled systems, LacI overexpression as with the *lacI*^*q *^phenotype is thus mandatory (either from the chromosome or the plasmid) to prevent such deletion.

Even though the double *lac *operator was exploited by the cell as the homologous region for recombination, the underlying problem is the extremely strong selective pressure, not the opportunity. Even in the absence of this particular homology, deletion between imperfect homologous regions or induction of an early frameshift in tracts of one particular base [38,39] are two efficient mechanisms that would cause the same kind of loss of expression. Therefore, the pragmatic solution to such a problem must lie in the supply of sufficient repressor, and not in the removal of the second binding site. We further note that the expression system is extremely robust in the presence of a *lacI*^*q*+ ^phenotype, from micro scale to fermentation.

In the past few years, paralleling the rapid progress in the availability of complete genome sequences and thus the identification of novel ORFs, numerous techniques for automated and manual high-throughput protein expression and purification have been developed [47-53]. It is commonly believed that modern expression vectors, especially commercial and widely used ones, have been optimized, e.g. to prevent the collision of transcription and replication machinery [12], and that any problem would at least result in a clear phenotype. In other words, such malfunction of a given expression vector would be expected not to stay unnoticed even in parallel or automated processes. However, in such high-throughput protein production approaches, one would ironically lose the best behaving and most robust proteins. Similarly, in fermentation processes with very long induction times, the occurrence of such deletions cannot per se be excluded. The complete absence of protein expression, without indication of plasmid loss or easily detectable large rearrangements, may thus warrant a more detailed investigation of the problem. Additionally, full repression even in the absence of a visible problem, is a useful precaution.

## Methods

Unless stated otherwise, all molecular biology experiments were performed according to protocols of Sambrook and Russell [54].

### Strains

The strains used in this study were *Escherichia coli *XL1 blue F' [30] (*recA*, *endA1*, *gyrA96*, *thi*, *hsdR17 *(r_K_^-^, m_K_^+^), *supE44*, *relA1*, *λ*^-^, *lac*^-^, [F'::*Tn10(tet)*, *proA*^+^*B*^+^, *lacI*^*q *^*Z*ΔM15]), RV308 [32], (*Δ(lac)χ74 galPO-308*::IS2 *rpsL*), DH5α [33] (*F*^-^, *endA1 hsdR17(r*_*K*_^-^*m*_*K*_^+^) *supE44 thi-1 λ*^-^*recA1 gyrA96 relA1 deoR, Δ(lacZYA-argF)U169, φ80dlacZΔM15*), DH5αZ1 [31] (DH5α *lacI*^*q*^*tetR*^+^), and SB536 [34] (w.t. strain WG1 Δ*fhuA ΔhhoAB*). The stocks of the strains were stored in 20% (v/v) glycerol solution at -80°C.

### Plasmids

Plasmids used in this study are listed in Table 1 in the Additional file 1. The sequences of all inserts in plasmids that were generated by PCR were confirmed by sequencing.

All cloning and plasmid preparation for subsequent experiments was performed using *E. coli *XL1 Blue F' (Stratagene, USA). The plasmid preparation for the comparative expression experiments in different strains started from a single colony that was then grown in LB medium supplemented with 50 μg/ml ampicillin under glucose repression (1% (w/v)) to give a "master" plasmid preparation. This DNA was than used in all subsequent transformations.

### Transformation efficiency and comparative expression experiments

Transformation efficiency was determined by introducing a normalized amount of the various expression plasmids prepared as described above into the *E. coli *test strains. This was done by incubation of the normalized plasmid solution with 30 μl of a competent cell solution [55] of the respective expression strains for 20 min on ice. Afterwards this mixture was directly plated, without further outgrowth in liquid media, on LB agar plates containing 1% (w/v) glucose and 50 μg/ml of ampicillin. Cells were grown at 37°C overnight.

For small scale expression tests single clones from these plates were taken to inoculate fresh LB medium supplemented with 1% (w/v) glucose (repression conditions) and 50 μg/ml ampicillin and grown up under vigorous shaking at 37°C. Protein expression was induced at an OD_600 _of 0.5–0.8 by the addition of 1 mM IPTG. After 4 hours, aliquots of the samples were withdrawn, normalized to OD_600 _and cells were disrupted by heating at 95°C for 15 minutes in SDS-loading buffer. Proteins were subsequently separated by SDS-PAGE.

### Identification of 19 kDa protein

Prior to N-terminal sequencing cell samples were normalized to cell density and proteins were separated by standard 15% SDS-PAGE. Subsequently samples were transferred to an Immobilon-P membrane (Millipore, Billerica, MA, USA) using semi-dry electroblotting [56]. Proteins were stained with Coomassie Brilliant Blue, respective protein bands of interest were cut out and subjected to Edman-degradation combined with HPLC analysis of the degradation products. The result of the HPLC analysis was subjected to the SIB BLAST Network Service [57].

## Competing interests

The authors declare that they have no competing interests.

## Authors' contributions

UH made the initial observation of complete lack of production in *E. coli *RV308 and provided this strain for testing. MAK conceived of the study, carried out the experiments and drafted the manuscript. APL conceived of the study, participated in its design, and drafted the manuscript. All authors read and approved the final manuscript.

## Supplementary Material

Additional file 1 **Supplementary material. **Description of the expression plasmids used and methods used in determining transformation efficiency and comparative expression experiments. Click here for file
