# Supplementary material for: Facile promoter deletion in Escherichia coli in response to leaky expression of very robust and benign proteins from common expression vectors
Source: Microb Cell Fact. 2009 Jan 26;8:8. doi: 10.1186/1475-2859-8-8 (PMC2655282; doi:10.1186/1475-2859-8-8)
Supplement: Additional file 1 — Supplementary material. Description of the expression plasmids used and methods used in determining transformation efficiency and comparative expression experiments. [file 1475-2859-8-8-S1.pdf]

Supplementary Material:
Facile promoter deletion in *Escherichia coli* in response to leaky expression of very robust and benign proteins from common expression vectors

Martin Kawe[1], Uwe Horn[2] and Andreas Plückthun[1*]

[1]Biochemisches Institut, Universität Zürich, Winterthurerstrasse 190, CH-8057 Zürich, Switzerland. [2]Leibniz Institute for Natural Product Research and Infection Biology — Hans-Knöll-Institute

[*]Corresponding author

Expression plasmids used

We used a plasmid expression system with well established components, closely analogous to the widely employed pQE30 backbone (a commercial vector from Qiagen). As shown in Figure S1, it consists of the strong inducible T5 promoter under control of two *lac*-operator sequences in series [1], the ORF of the protein of interest, followed by two terminators in series (a T7 transcription terminator and a strong *rrnB* t_1 transcription terminator [2]), a ColE1 origin of replication (as present in pBR322, but without the *rop* gene [3]), the β-lactamase gene under its own promoter to confer resistance to ampicillin with the strong transcription terminator t_0 of phage λ, and, facultatively, the *lacI* gene coding for the *lac* repressor, which is under control of the *lacI*^{q} promoter and terminated by the strong t_{HP} terminator [4], for tight regulation of the operon. The expression vectors used (summarized in Table S1) are called pMPAG77 (without *lacI* gene cassette), and pMPAG6 (carrying a *lacI* gene cassette under the *lacI*^{q} promoter, this being the only difference to pMPAG77).

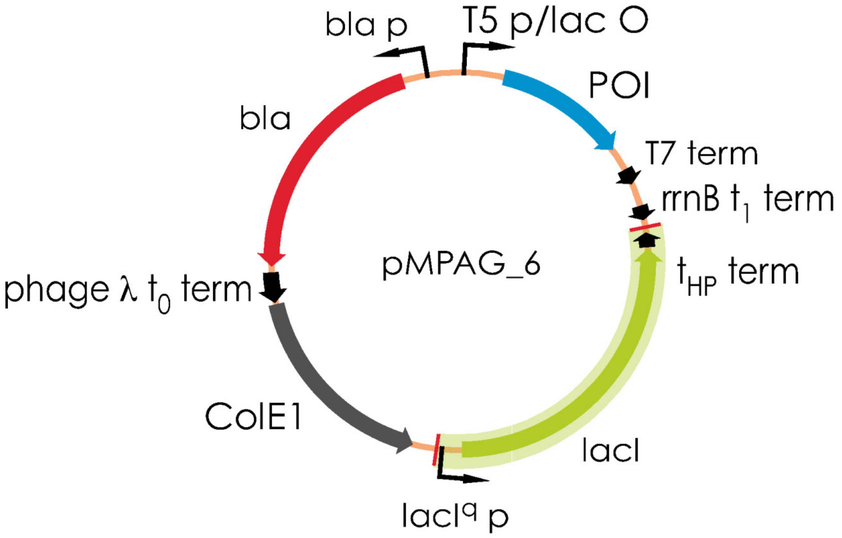

Figure S1: Schematic representation of the two expression vectors used. Both contain a β-lactamase gene to confer resistance to ampicillin (red) with phage λ t_0 terminator, a ColE1 origin of replication (grey), a strong T5 promoter under control of two *lac* operators in series (see Figure 2), the protein of interest (POI, here either DARPins G3 or E3_5, MPB, gpD; light blue) with T7 and *rrnB* t_1 terminators in series, and in case of the pMPAG6-vector type, a *lacI* gene under control of the *lacI^q* promoter (green) with t_{HP} terminator (P. Forrer et al., unpublished). In pMPAG77-type this *lacI* gene cassette is lacking (highlighted by the area in light green, flanked by two red bars indicating the restriction sites used to remove this gene cassette).

Table S1 - Plasmids

Plasmid	Relevant details	Source/Reference
pMPAG6	Ap^R, ColE1, expression vector, *lac*I^{q+}, proteins of interest (POI) are under control of $P_{T5/lac}$	P. Forrer[1] et al., unpublished
pMPAG77	Constructed by cutting out the *lacI*^{q} gene from pMPAG_6 with restriction enzymes *Xba*I, *Avr*II and *Xcm*I and subsequent religation of the vector backbone.	this work
pMPAG77_ STOP	Ap^R, ColE1; *lac*I^{q-}, encodes a DARPin sequence under control of $P_{T5/lac}$ which is terminated by a STOP codon after the fifth amino acid position	this work
pAT187_MBP	Ap^R, ColE1, RGS-His_6-tag-MBP under control of $P_{T5/lac}$, *lac*I^{q-}; identical to the pMPAG_77 series except for a nonfunctional chloramphenicol acetyl transferase gene after the POI	P. Forrer, unpublished

Ap^R Ampicillin resistance, POI protein of interest.

[1]Molecular Partners AG; Schlieren/CH

pMPAG77_STOP is a derivative of pMPAG6_her2-G3. The modified ORF contains a STOP codon at the fifth amino acid position. It was used for the expression of the first N-terminal amino acids of the DARPin G3 under control of a T5/lac promoter. The plasmid carries no *lac*I gene. It was constructed by site directed mutagenesis with primers MAK_DpnI_#6F (5'- GATCGCATCA CCATCACTGA CACGGATCCG ACCTGGG -3') and MAK_DpnI_#6R (5'- CCCAGGTCGG ATCCGTGTCA GTGATGGTGA TGCGATC - 3') following the QuikChange™ protocol of Stratagene® (La Jolla, CA).

Transformation efficiency and comparative expression experiments.

Transformation efficiency was determined by introducing a normalized amount of the various expression plasmids prepared as described in Materials and Methods into the *E. coli* test strains which were displaying a *lacI*^{q} (Figure S2, straight black line with red box, symbolizing the chromosome or an F-plasmid) or *lacI*$^{q-}$ (i.e., either *lacI*$^{-}$ or *lacI*^{wt}) (straight black line without red box) phenotype. The tested expression vectors were harboring or lacking a *lacI* gene under *lacI*^{q} control (red segment on plasmid; "+" and "–" signs denote the presence of chromosomal or plasmid-encoded LacI overexpression, respectively). We observed that the transformation efficiency in a double *lacI*$^{q+}$ background was indistinguishable from cells displaying a single *lacI*$^{q+}$ background (*lacI*^{q} either provided on the genome or from the plasmid). In a double *lacI*$^{q-}$ background, transformation efficiency was only slightly lower for three out of the four proteins tested, compared to the other combinations. Only when protein D [5] was expressed was there a clear reduction in cfu in the double *lacI*$^{q-}$ background. The observed reduction in colony number for all other strains harboring the remaining proteins was still in the range of variation for different preparations of competent cells and thus the collateral deletion leading to non expression of our test proteins would have stayed unnoticed without the respective controls.

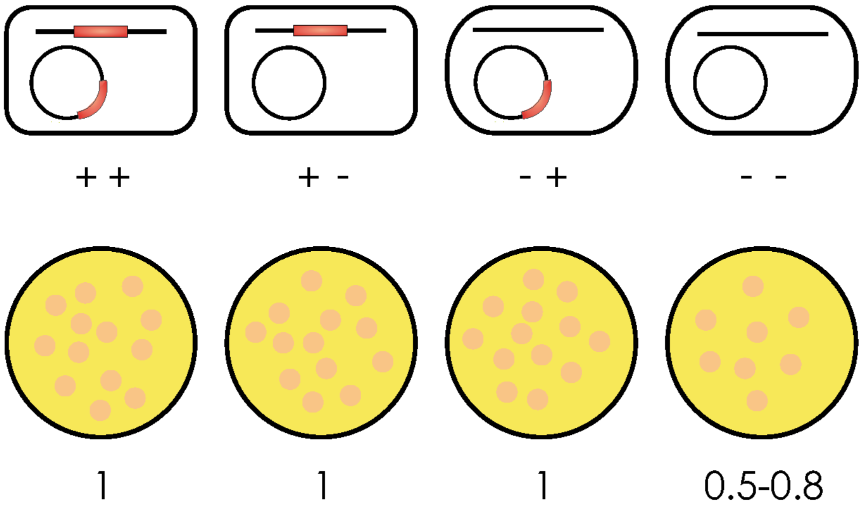

Figure S2: Schematic Depiction of transformation efficiency

References

1. Bujard H, Gentz R, Lanzer M, Stueber D, Mueller M, Ibrahimi I, Haeuptle MT, Dobberstein B: **A T5 promoter-based transcription-translation system for the analysis of proteins *in vitro* and *in vivo***. *Methods Enzymol* 1987, **155**:416-433.

2. Artsimovitch I, Svetlov V, Anthony L, Burgess RR, Landick R: **RNA polymerases from Bacillus subtilis and Escherichia coli differ in recognition of regulatory signals in vitro**. *J Bacteriol* 2000, **182**:6027-6035.

3. Balbas P, Soberon X, Merino E, Zurita M, Lomeli H, Valle F, Flores N, Bolivar F: **Plasmid vector pBR322 and its special-purpose derivatives--a review**. *Gene* 1986, **50**:3-40.

4. Nohno T, Saito T, Hong JS: **Cloning and complete nucleotide sequence of the Escherichia coli glutamine permease operon (glnHPQ)**. *Mol Gen Genet* 1986, **205**:260-269.

5. Forrer P, Jaussi R: **High-level expression of soluble heterologous proteins in the cytoplasm of Escherichia coli by fusion to the bacteriophage lambda head protein D**. *Gene* 1998, **224**:45-52.
